# Calcium Ions Signaling: Targets for Attack and Utilization by Viruses

**DOI:** 10.3389/fmicb.2022.889374

**Published:** 2022-07-04

**Authors:** Yang Qu, Yingjie Sun, Zengqi Yang, Chan Ding

**Affiliations:** ^1^College of Veterinary Medicine, Northwest A&F University, Yangling, China; ^2^Department of Avian Infectious Diseases, Shanghai Veterinary Research Institute, Chinese Academy of Agricultural Science, Shanghai, China; ^3^Jiangsu Co-innovation Center for Prevention and Control of Important Animal Infectious Diseases and Zoonoses, Yangzhou, China

**Keywords:** virus, calcium homeostasis, calcium channels, calcium pumps, cell death, innate immune, antiviral responses

## Abstract

Calcium, as a second intracellular messenger, participate in various physiological and biochemical processes, including cell growth and proliferation, energy metabolism, information transfer, cell death, and immune response. Ca^2+^ channels or pumps in plasma and organelle membranes and Ca^2+^-related proteins maintain Ca^2+^ homeostasis by regulating Ca^2+^ inflow, outflow and buffering to avoid any adverse effects caused by Ca^2+^ overload or depletion. Thus, Ca^2+^ signaling also provides a target for virus invasion, replication, proliferation and release. After hijacking the host cell, viruses exploit Ca^2+^ signaling to regulate apoptosis and resist host immunity to establish persistent infection. In this review, we discuss cellular Ca^2+^ signaling and channels, interaction of calcium-associated proteins with viruses, and host cell fate, as well as the role of Ca^2+^ in cell death and antiviral response during viral infection.

## Introduction

Calcium is an important trace element in animals and is an initiator and regulator of a variety of intra- and extracellular pathways involved in several physiological activities, including heartbeat, muscle contraction, brain memory storage, and neurosecretory system signaling. Ca^2+^ are involved in various intracellular physiological and biochemical processes as an intracellular second messenger, maintaining cell growth and proliferation, energy metabolism and information transfer (Berridge et al., 1998; Contreras et al., 2010). Under normal physiological conditions, intracellular Ca^2+^ homeostasis is vital for cells. Different concentrations of Ca^2+^ exist between organelles to form a large Ca^2+^ gradient. To maintain normal cellular activities, Ca^2+^ present in each cellular compartment use special channels and pumps to maintain dynamic equilibrium through influx, efflux, buffering and storage. The endoplasmic reticulum (ER) acts as a cellular Ca^2+^ store to regulate cellular Ca^2+^ homeostasis. The mitochondria are Ca^2+^-buffering organelles that maintain Ca^2+^ homeostasis by absorbing and releasing Ca^2+^ to establish contacts with the cytoplasm and other organelles in response to various intracellular signals (Tang et al., 2015; Panda et al., 2021).

When cells are stimulated by exogenous factors or endogenous disruption of structural function, an imbalance in Ca^2+^ homeostasis is inevitably induced and accompanied by an elevated intracellular Ca^2+^ concentration (Pinton et al., 2008). This pathological increase in Ca^2+^ arises from elevated inward flow from the extracellular environment and massive release of intracellular Ca^2+^ stores. Normal transient stimuli cause an increase in Ca^2+^ concentration that enhances cellular metabolism and promotes ATP production, whereas large and persistent calcium overload causes ER and mitochondrial stress, activating intracellular enzyme cascades, and sequentially triggering the death process (Pinton et al., 2008; Di Benedetto et al., 2013).

Viruses are noncellular organisms that parasitize living cells and replicate using the material and energy of host cells. After hijacking the host cell, viruses rapidly exploit various host cell systems. Viruses disrupt intracellular Ca^2+^ homeostasis by adapting or inhibiting Ca^2+^ signaling pathways and other Ca^2+^-dependent processes, creating favorable conditions for maximum utilization of host cell resources and replication of progeny viruses (Zhou et al., 2009; Panda et al., 2021). Ca^2+^ plays a role in almost every step of the viral replication cycle, includes virus entry, protein expression and modification, and virion maturation and release. The interaction between viruses and Ca^2+^ falls into three main categories: (1) viral proteins directly or indirectly disrupt intracellular Ca^2+^ homeostasis by modulating calcium channels and pumps or host membrane permeability; (2) Ca^2+^-regulated proteins or Ca^2+^-dependent pathways are involved in regulation of the virus life cycle; and (3) viral proteins bind directly to Ca^2+^ to hijack the host and destroy the integrity of cellular structure and function (Zhou et al., 2009).

What is the consequence of Ca^2+^ alterations in the life cycle of host cells during viral infection? First, the moderate increase in Ca^2+^ concentration caused by viral infection induces Ca^2+^-dependent enzymatic processes or activation of Ca^2+^-sensitive transcription factors to promote viral replication and persistent infection (Bergqvist and Rice, 2001; Bergqvist et al., 2003; Zhou et al., 2009). Second, Ca^2+^ plays a crucial role in the initiation and effectuation of cell death. Various stress injuries caused by viral infections induce Ca^2+^ overload in mitochondria, leading to mitochondrial membrane collapse, energy metabolism disorders, and ultimately cell death, such as necrosis, apoptosis, and autophagic cell death (Raffaello et al., 2016; Bravo-Sagua et al., 2017). At the same time, the host also mobilizes Ca^2+^ signaling to initiate an antiviral response to resist virus invasion (Crabtree and Clipstone, 1994; Tano and Vazquez, 2011; Mathavarajah et al., 2019). The interaction between Ca^2+^ signals and different pattern recognition receptor (PRR) signals resists exogenous pathogenic challenges and endogenous danger signals (Kong et al., 2021). This review highlights viral disruption of cellular Ca^2+^ signaling networks by interacting with host calcium pathways to promote self-replication and persistent infection, and the role of Ca^2+^ signaling networks in regulating cell death and antiviral responses.

## Cellular Ca^2+^ Channels and Pumps: Regulatory Targets for Virus Multiplication

In the normal state, the extracellular concentration of Ca^2+^ is up to the millimolar level. The cytoplasmic Ca^2+^ concentration is maintained at >100 nM, while the concentration in the ER or sarcoplasmic reticulum (SR), as the largest intracellular Ca^2+^ storage organelle, is several hundred micromolar (Berridge et al., 2003; Rizzuto et al., 2012; Zampese and Pizzo, 2012; Raffaello et al., 2016). Viruses take advantage of the large cellular Ca^2+^ concentration in different organelles to regulate cellular calcium signaling through promoting ATP synthesis, accelerating some Ca^2+^-dependent enzymatic processes and upregulating Ca^2+^-sensitive transcriptional factors. Viruses hijack and rely on the Ca^2+^ signaling networks to facilitate their penetration, replication, assembly and export to establish sustained infection.

**Figure 1:**
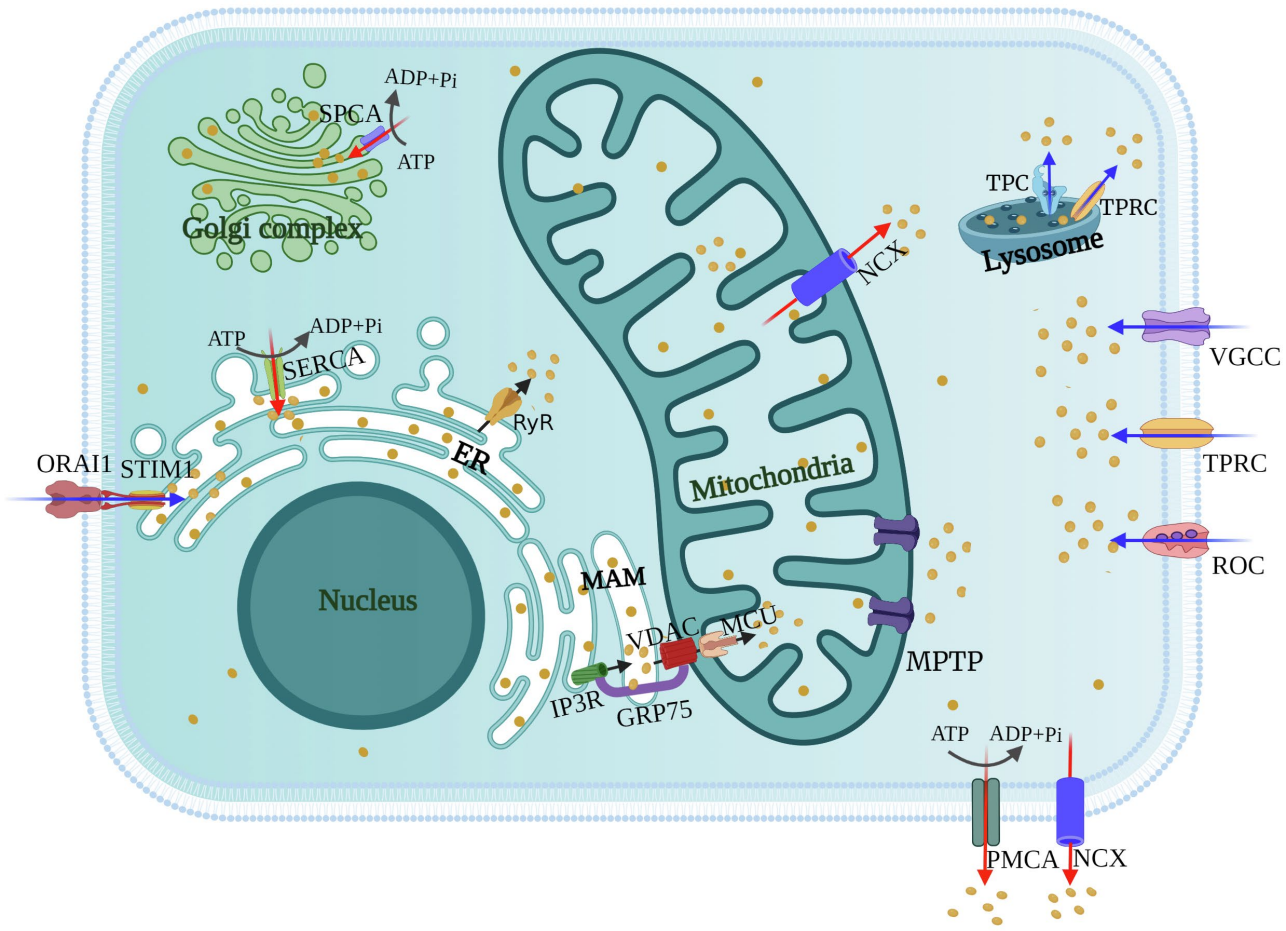
Schematics of cellular calcium channels and pumps. Calcium channels [voltage-gated calcium channels (VGCCs), receptor-operated calcium channels (ROCs), store-operated Ca^2+^ channels (SOCs), and transient receptor potential (TRP) channels] mediate the entry of Ca^2+^ from the extracellular environment or the release of Ca^2+^ from the Lysosome (blue arrows). The inositol trisphosphate receptors (IP_3_Rs) and ryanodine receptors (RyRs) on the ER or Golgi regulate the release of Ca^2+^ from intracellular stores (black arrows). Ca^2+^ is transported between the ER and mitochondria *via* mitochondrial-associated membranes (MAMs), and mitochondrial uptake of Ca^2+^ is through voltage-dependent anion channels (VDACs) and mitochondrial calcium uniporters (MCUs). Calcium pumps [SR Ca^2+^-ATPase (SERCA), secretory pathway Ca^2+^-ATPase (SPCA), and plasma membrane Ca^2+^-ATPase (PMCA)] and the Na^+^/Ca^2+^ exchanger (NCX) transport Ca^2+^ from the cytosol to extracellular environment or intracellular stores (red arrows).

As the first barrier against viruses, the plasma membrane has a variety of ion channels involved in the exchange of substances inside and outside the cytoplasm. Cellular maintenance of Ca^2+^ homeostasis requires regulation of calcium channels and pumps on the plasma membrane. During their life cycle, viruses utilize various calcium channels and pumps of the host to resist the membrane barriers to create favorable conditions for themselves. Host Ca^2+^ channels [voltage-gated calcium channels (VGCCs), receptor-operated calcium channels (ROCs), and store-operated calcium channels (SOCs)] mediate movement of Ca^2+^ across the plasma membrane and entry of Ca^2+^ from the extracellular medium, while the plasma membrane Ca^2+^-ATPase (PMCA) and the Na^+^/Ca^2+^ exchanger (NCX) extrude Ca^2+^ from the cell (Zhou et al., 2009; Figure 1). In this section, we discuss the significance of cellular Ca^2+^ signaling, channels and pumps in the viral life cycle during virus-host conflict.

**Table 1:** Ca^2+^ channels and pumps used as targets by viruses.

Calcium channels or pumps	Virus and viral proteins	Consequences of interaction	References
Voltage-gated calcium (VGC) channels	IAV HA	HA binds to Ca_V_1.2 to promotes virus entry	Fujioka et al., 2018
	HIV gp120/Tat	gp120/Tat activate VGCC to promotes extracellular calcium influx	Holden et al., 1999; Mayne et al., 2000
	Flavivirus (JEV, ZIKV, DENV, WNV)	VGCC blockers inhibit flavivirus replication	Wang et al., 2017
	Rotavirus	Rotavirus infection of cells activates a cation channel	Pérez et al., 1999
	HSV	HSV-1 downregulates the Cav3.2 channel to escape host detection	Zhang et al., 2019
	EBV/CMV	L-type Ca^2+^-channel blockers inhibit the increase in intracellular Ca^2+^ by virus	Nokta et al., 1987; Dugas et al., 1988
Store-operated calcium (SOC) channels STIM1/ORAI1	DENV, EBOV, MARV, JUNV	Virus activate STIM1 and ORAI1 channel to trigger host cell Ca^2+^ signals, promoting virion assembly and budding	Han et al., 2015; Dionicio et al., 2018
	HBV X proteins	HBx protein directly binds and modifies STIM1-ORAI1 complexes to regulate Ca^2+^	Yao et al., 2018
Transient receptor potential (TRP) channels	RSV, MV, HRV	Viruses upregulated TRP channels like TRPV1, TRPA1 and TRPM8 to create an intracellular Ca^2+^ environment conducive to their replication	Abdullah et al., 2014; Omar et al., 2017
	ZIKV, DENV, HCV	TRPV4 drives DDX3X nuclear translocation and activated DDX3X-dependent functions to promote the viral RNA metabolism	Doñate-Macián et al., 2018
Receptor-operated calcium (ROC) channels	ZIKV, JEV	Viruses use NMDAr to induce neuronal cell death and inflammatory response	Sirohi and Kuhn, 2017; Chen et al., 2018
IR3Rs or RyRs	HBV, HTLV-1, PV, HSV, IAV	Viruses induce increased cytoplasmic Ca^2+^ from ER/SR by enhancing IP3Rs or RyRs activity	Hartshorn et al., 1988; Ding et al., 2002; Cheshenko et al., 2003; Xia et al., 2006; Brisac et al., 2010
Mitochondrial calcium channels	HIV protein R	protein R locates at mitochondria and cooperates with the ANT, leading to the release of Ca^2+^ in mitochondria	Jacotot et al., 2000
	HBV	HBx interacts with VDAC to trigger mitochondrial Ca^2+^ release	Chami et al., 2003
	HCV	HCV core protein triggers influx of Ca^2+^ to mitochondria *via* MCU	Ivanov et al., 2015
	PV, CV	Viruses induce Ca^2+^ uptake by MCU and VDAC of mitochondria	Brisac et al., 2010; Peischard et al., 2019
	MCMV, JEV, IAV, KHSV	The opening of MPTP release of Ca^2+^ from the mitochondria	Feng et al., 2002; Chanturiya et al., 2004; Huang et al., 2016; Panel et al., 2019
Two-pore channels (TPCs)	EBOV, MERS, MCPyV, SV40	Viruses mobilize Ca^2+^ from the lysosomal stores through TPC channels to facilitate virus–endosome membrane fusion	Sakurai et al., 2015; Gunaratne et al., 2018; Dobson et al., 2020
Calcium pumps	DENV, WNV, ZIKV	Ca^2+^ are pumped into Golgi by SPCA1 and trigger to produce functional viral glycoproteins	Hoffmann et al., 2017
	RSV	SERCA induce Ca^2+^ returning to the ER from the cytosol to promote viral genome replication and/or transcription	Cui et al., 2016

### Adsorption, Penetration, and Uncoating of the Virus

**Figure 2:**
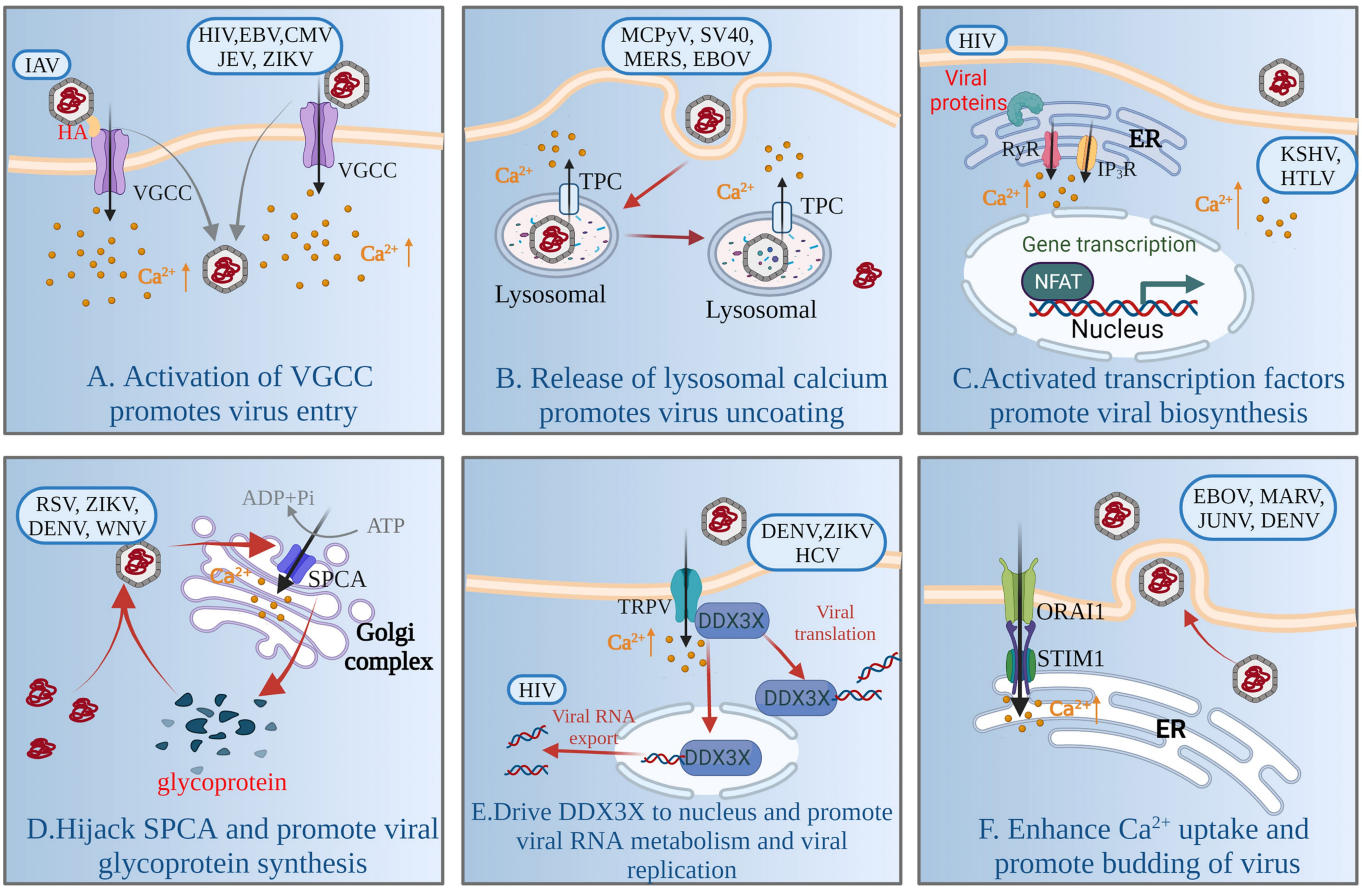
Model of Ca^2+^ signaling network involved in virus replication. By activating VGCC, the virus induces influx of extracellular Ca^2+^ and promotes adsorption and entry of virions **(A)**. Viruses rely on TPC activity to mobilize Ca^2+^ from lysosomes and to degrade viral capsids through the lysosomal network **(B)**. Viral infection increases cytoplasmic Ca^2+^, promotes activation of calcium-sensitive transcription factors (NFAT) and coactivators (P300), and induces viral RNA transcription and establishment of persistent infection **(C)**. Viruses enhance SPCA and pump Ca^2+^ into the Golgi complex to promote synthesis of functional viral glycoproteins **(D)**. Viruses activate TRPV-mediated Ca^2+^ influx and drive DDX3X nuclear translocation, promoting viral RNA metabolism and viral replication **(E)**. Viruses activate the SOCE channel or promote interaction between STIM1 and ORAI1, enhancing cellular Ca^2+^ uptake and promoting budding of mature virus particles **(F)**. For a complete list of definitions, see Table 1.

Adsorption, penetration and uncoating are the first stage of viral replication. Viruses encode multiple proteins to manipulate the plasma membrane Ca^2+^ channels [VGCCs and two-pore channels (TPCs)] involved in regulating cellular Ca^2+^ uptake to increase intracellular Ca^2+^, thereby facilitating viral entry and replication. For example, human immunodeficiency virus type 1 (HIV-1) gp120 and Tat protein, and porcine rotavirus (RV), activate VGCCs to increase the levels of intracellular Ca^2+^ (Holden et al., 1999; Pérez et al., 1999; Mayne et al., 2000). The glycoprotein hemagglutinin (HA) of Alphainfluenzavirus influenzae (IAV) binds to voltage-dependent Ca^2+^ channel Ca_v_1.2, triggering intracellular Ca^2+^ increase, and activates endocytosis to gain entry into cells (Fujioka et al., 2013, 2018; Figure 2A). Human alphaherpesvirus 1 (HSV)-1 infection results in a significant decrease in protein expression of Ca_v_3.2 T-type Ca^2+^ channel subunit to escape detection by host cells (Zhang et al., 2019). Some VGCC blockers inhibit viral replication by inhibiting intracellular Ca^2+^ increase, such as Human gammaherpesvirus 4 (EBV), Human betaherpesvirus 5 (CMV), and flaviviruses [such as Japanese encephalitis virus (JEV), Zika virus (ZIKV), dengue virus (DENV) and West Nile virus (WNV)] (Nokta et al., 1987; Dugas et al., 1988; Wang et al., 2017). Intracellular Ca^2+^ oscillations are the trigger for viral penetration or uncoating (Baravalle et al., 2004; Danta, 2020). For porcine rotavirus, the critical step for uncoating and membrane permeabilization is a decrease in Ca^2+^ concentration of cytosol accompanying dissociation of viral Ca^2+^-stabilized proteins (Chemello et al., 2002; Salgado et al., 2018). The structure of some viruses contains a flexible surface loop that binds divalent Ca^2+^, and have been found to be essential for virus infectivity (Simpson et al., 2000).

In the 1990s, the role of the endolysosomal system for calcium storage was discovered (Genazzani and Galione, 1996). The calcium exchange and Ca^2+^-mediated functional coupling also exists at the lysosome-ER interface (Raffaello et al., 2016). The mucolipin family of transient receptor potential (TRPML) channels and TPCs are involved in the release of Ca^2+^ from lysosomes (Patel et al., 2011; Figure 1). Many viruses mobilize Ca^2+^ from the endolysosomal stores through TPCs, and some viruses are dependent on the activity of TPCs to enable capsid entry into cells using endolysosomal networks, such as Alphapolyomavirus quintihominis (MCPyV), Betapolyomavirus macacae (SV40), Middle East respiratory syndrome coronavirus (MERS-CoV) and Zaire Ebola virus (EBOV; Sakurai et al., 2015; Gunaratne et al., 2018; Dobson et al., 2020; Figure 2B).

### Biosynthesis of the Virus

Viral biosynthesis includes mRNA transcription, and protein and DNA or RNA synthesis and metabolism, in which Ca^2+^-signaling networks play an important role. Some viruses or viral proteins are involved in increasing cytoplasmic Ca^2+^, leading to activation of the Ca^2+^-sensitive nuclear factor of activated T cells (NFAT) to support viral RNA transcription and establish persistent infection (Figure 2C). For example, HIV accessory protein Nef cooperates with Vpr to trigger release of ER Ca^2+^ that leads to activation of NFAT (Kinoshita et al., 1997; Lahti et al., 2003). Primate T-lymphotropic virus 1 (HTLV)-1 regulatory and accessory genes p12^I^ activates NAFT (Ding et al., 2002), and upregulates expression of another Ca^2+^-sensitive transcription factor, P300 (Nair et al., 2006). Human gammaherpesvirus 8 (KHSV) K1 protein, is a transmembrane glycoprotein, increased cellular tyrosine phosphorylation and intracellular Ca^2+^ mobilization, activating NFAT and AP-1 transcription factor and producing inflammatory cytokines to promote viral dissemination (Lee et al., 2005). Furthermore, intracellular calcium triggers calcineurin-dependent signal transduction resulting in reactivation of latent KHSV infection (Zoeteweij et al., 2001).

Viruses directly activate calcium channels and pumps on some organelles to promote viral protein synthesis and modification. There are reports that glycoproteins of Paramyxoviridae, Flaviviridae and Togaviridae families fail to mature in SPCA1-deficient cells, Ca^2+^ is pumped into the Golgi by SPCA1, which triggers synthesis of functional viral glycoproteins that are essential for viral spread (Hoffmann et al., 2017; Figure 2D). Some respiratory viruses, such as Human orthopneumovirus (RSV), Measles morbillivirus (MV) and Human rhinovirus A, promote upregulation of TRP channels such as TRPV1, TRPA1 and TRPM8, and use TRP channels to create an intracellular Ca^2+^ environment conducive to their replication (Abdullah et al., 2014; Omar et al., 2017). DENV, hepacivirus C (HCV) and ZIKV or the purified viral envelope protein activate TRPV4-mediated Ca^2+^ influx that drives DDX3X nuclear translocation to promote viral replication; what’s more, TRPV4-DDX3X interaction regulates the nuclear export and translation of unspliced HIV-1genomic RNA (gRNA) and viral RNA metabolism (Doñate-Macián et al., 2018; Figure 2E).

Viral proteins can also bind directly to Ca^2+^ to promote viral biosynthesis. For example, Bluetongue virus (BTV) nonstructural phosphoprotein NS2 is a dedicated Ca^2+^-binding protein that significantly enhances NS2 phosphorylation, triggering viral inclusion body formation and facilitating viral replication and assembly (Rahman et al., 2020). High levels of cytosolic Ca^2+^ facilitates hepatitis B virus (HBV) core assembly, which is regulated by the viral multifunctional regulatory protein HBx (Choi et al., 2005). High Ca^2+^ levels promote Ca^2+^-dependent activation of proline-rich tyrosine kinase 2 and focal adhesion kinase; and induce NFAT expression, which supports HBV reverse transcription and DNA replication (Lara-Pezzi et al., 1998).

### Maturation and Release of Virus

Most nonenveloped viruses release virus particles when the host cell is completely lysed, while enveloped viruses release virus particles by budding, which is accompanied by activation of Ca^2+^ channels and mobilization. SOCs mediated entry of Ca^2+^ is activated mainly by the membrane Ca^2+^ release-activated Ca^2+^ modulator 1(ORAI1) on the plasma membrane and stromal interaction molecule (STIM1) on the ER, which are stimulated by the depletion of internal Ca^2+^ stores. The budding process of enveloped viruses depends on the host Ca^2+^ signal mediated by SOCs (STIM1/ORAI1). Some viruses activate SOCs or depend on STIM1 and ORAI1 interaction to enhance cellular Ca^2+^ uptake for the budding of mature virus particles. Such viruses include DENV, EBOV, Marburgvirus (MARV) and Argentinian mammarenavirus (JUNV; Han et al., 2015; Dionicio et al., 2018; Figure 2F).

## Cellular Ca^2+^ Signaling Networks: A Life and Death Regulator of Viral Infection

**Figure 3:**
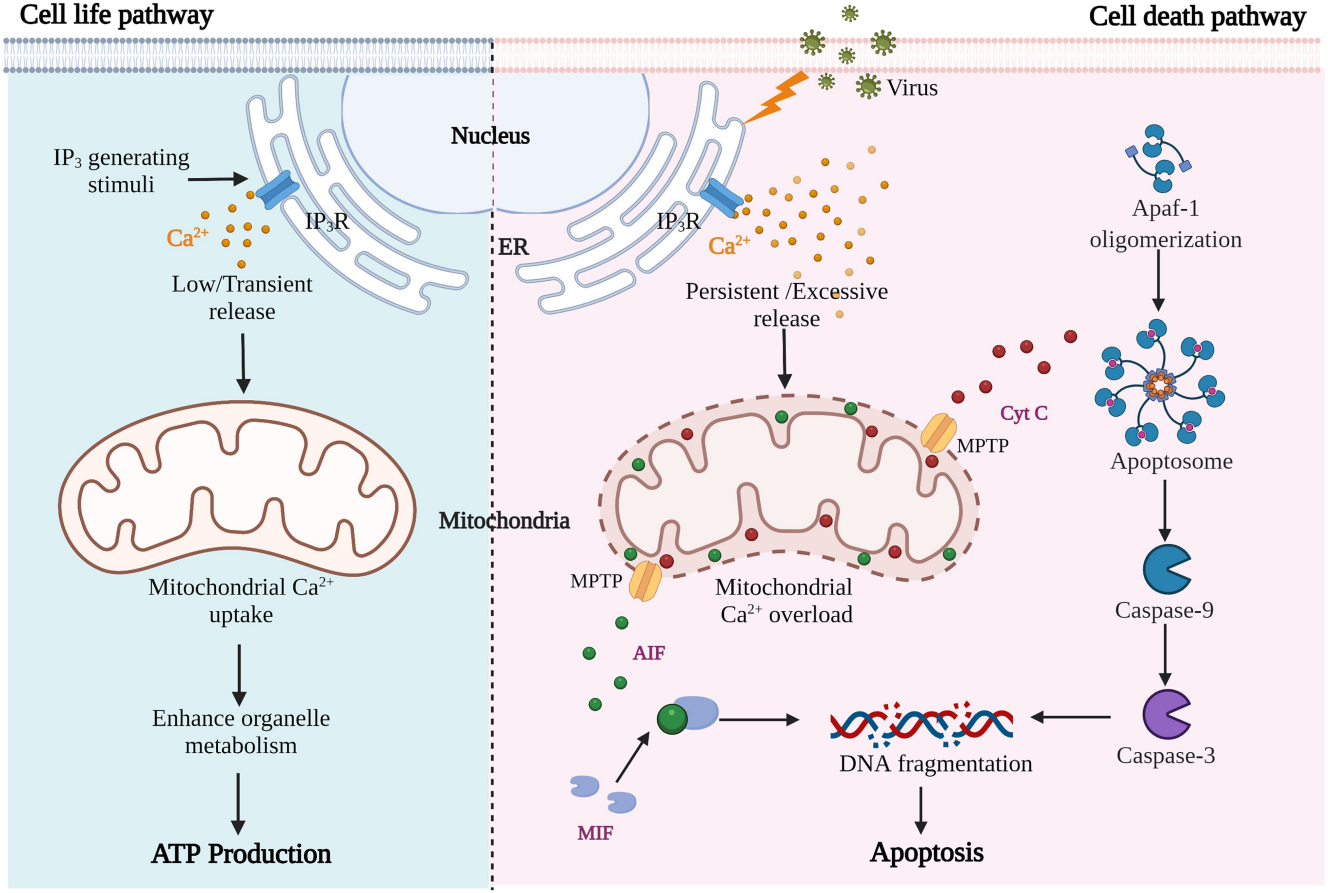
Results of cell metabolism or apoptosis induced by different Ca^2+^-related stimuli.

As previously mentioned, viral infection of cells almost inevitably leads to the production of pathological Ca^2+^ signals; the main source of which is increased extracellular entry and intracellular storage release. Mitochondria, as the center of cell survival and metabolism and buffer of Ca^2+^ signaling, participate in regulating energy synthesis, cell death, and other processes that determine cell life and death. Pathological Ca^2+^ accumulation and mitochondrial Ca^2+^ overload trigger the cell death process, including apoptosis or programmed cell death, necrosis, autophagic cell death and anoikis (Orrenius et al., 2003, 2015). Different states of abnormal Ca^2+^ homeostasis have different effects on cellular functions; low and transient stimulation enhances cellular metabolism and promotes ATP production, while large and continuous Ca^2+^ accumulation causes ER and mitochondrial stress, resulting in persistent mitochondrial damage and cell death (Pinton et al., 2008; Figure 3). Here, we focus on virus-induced mitochondrial Ca^2+−^overload-mediated apoptosis.

### Viral Infection in Ca^2+^-Mediated Apoptosis

Apoptosis is considered to be a form of cell suicide, regulated by its own process under physiological or pathological conditions. The morphological manifestations of apoptosis are cell shrinkage, nuclear fragmentation, chromatin condensation, and formation of apoptotic bodies. Ca^2+^ is an important signaling molecule that regulates apoptosis during viral infection. On the one hand, viral infection induces increased extracellular Ca^2+^ influx and Ca^2+^ release from intracellular storage, and the continuously increased Ca^2+^ initiates apoptosis. On the other hand, the release of Ca^2+^ from storage disrupts the stability of intracellular structure, and many key components of the apoptotic system are activated. These two forms often coexist in virus-induced apoptosis.

Mitochondria, as the core organelles of apoptosis, are hijacked and utilized by viruses. Viruses trigger apoptosis by increasing mitochondrial Ca^2+^ uptake, enhancing mitochondrial membrane permeability and promoting release of apoptotic factors. Cytochrome (Cyt) C, as an activator of the caspase family, is required for caspase-dependent apoptosis. It is released into the cytoplasm and binds to apoptotic protease activating factor (Apaf)-1 to form apoptosomes, triggering the caspase cascade *via* caspase-9 activation (Figure 3). For example, both HBV and HCV are hepatotropic viruses that cause chronic liver disease and hepatocellular carcinoma (Scrima et al., 2018), and they promote Ca^2+^ uptake in mitochondria and lead to reactive oxygen species (ROS) production and apoptosis (Li et al., 2007). During HBV infection, HBx protein interacts with VDACs to trigger the release of Cyt C from the mitochondria, which triggers apoptosis. Some inhibitors of Ca^2+^ channels can down-regulate the proliferation of virus and avoid the occurrence of apoptosis of host cells (Chami et al., 2003; Xia et al., 2006). In the same way, enteroviral infections cause high mitochondrial Ca^2+^ overload, mitochondrial dysfunction, and apoptosis. When treated with the BAPTA-AM, a Ca^2+^ chelating agent, viral replication was also inhibited along with the alleviation of apoptosis (Brisac et al., 2010; Peischard et al., 2019). Besides, some viral proteins, such as IAV PB1-F2, induce permeabilization and destabilization of mitochondrial membranes *via* changes in Ca^2+^ homeostasis, leading to macromolecular leakage and apoptosis (Chanturiya et al., 2004). Whereas HCV NS5A protein promotes IP_3_R degradation, inhibiting virus-induced apoptosis and establishing chronic infection (Kuchay et al., 2018). In this process, apoptosis appears to be a host suicide defense mechanism to prevent spreading of virus.

**Figure 4:**
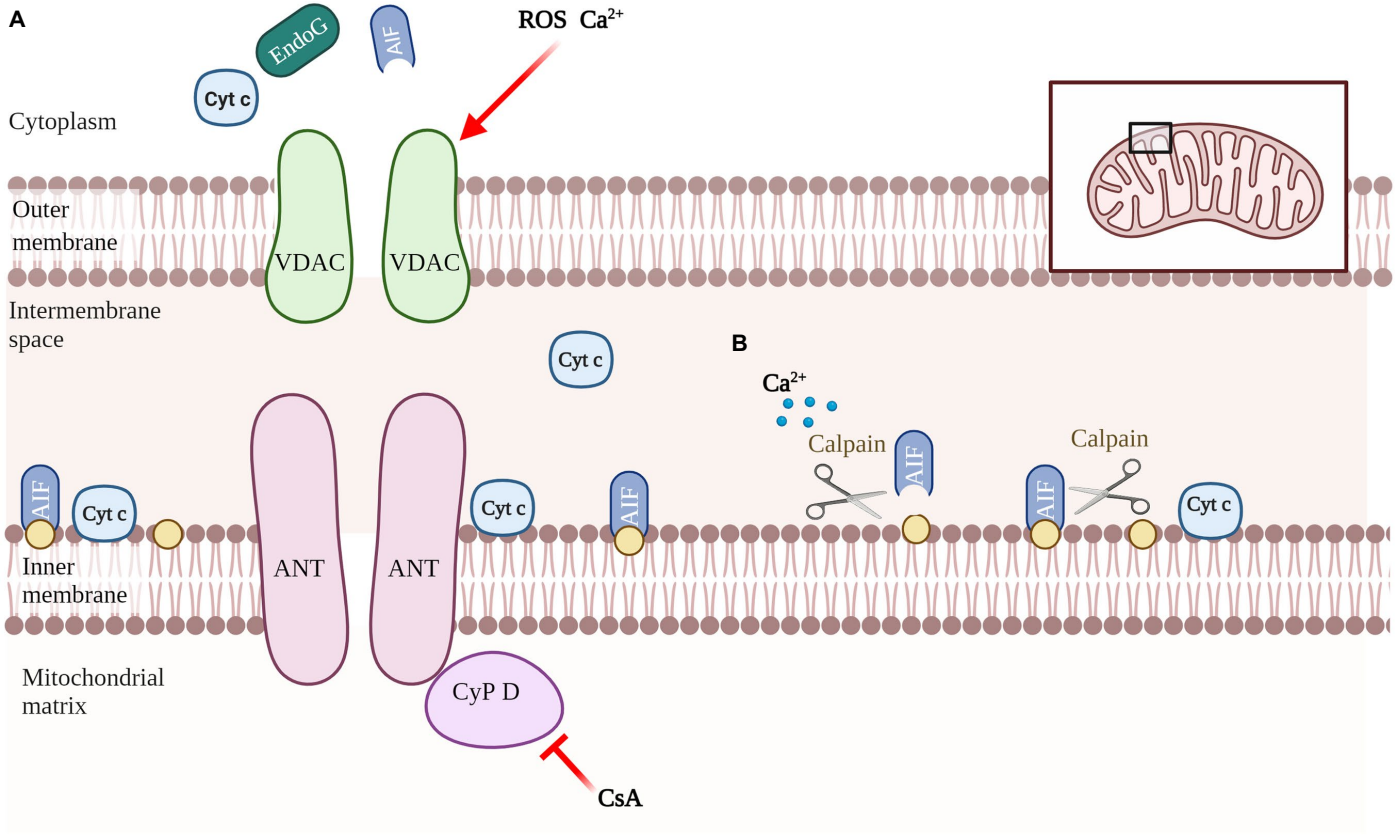
Schematic of mediating the release of apoptotic molecules. Composition of the nonspecific pore complex of MPTP, which triggers mitochondrial swelling and apoptotic molecules release. **(A)**. Involvement of calcium/calpain in AIF-activated processing **(B)**.

Another promoter of cell death is apoptosis-inducing factor (AIF), which mediates the regulation of caspase-independent apoptosis (Figure 3). AIF is a mitochondrial oxidoreductase with a molecular weight of about 62 kDa anchored to the inner mitochondrial membrane (IMM) in the vicinity of Complex I. when mitochondria are damaged and the mitochondrial permeability transition pore (MPTP) is open, activated AIF is released (Susin et al., 1996). Calpain is a calcium-dependent intracellular cysteine protease that cleaves mitochondrial AIF to promote AIF activation (Cao et al., 2007; Figure 4B). The released AIF recruits macrophage migration inhibitory factor (MIF) to the nucleus, fragmentating the DNA (Yu et al., 2002; David et al., 2009; Wang et al., 2016).

In conclusion, Ca^2+^ homeostasis imbalance, mitochondrial damage, and release of various apoptotic macromolecules are often associated with virus-induced apoptosis. From the perspective of virus, it exploits host resources by destroying intracellular Ca^2+^ homeostasis and regulates apoptosis to achieve the purpose of promoting its own replication. Similarly, apoptosis is also regarded by the host as an antiviral method, and the virus has achieved the establishment of persistent infection by regulating the activity of Ca^2+^ channels to prevent apoptosis.

### ER-Mitochondrial Calcium Disorder Mediated by Viral Infection

Under physiological conditions, mitochondrial Ca^2+^ uptake is thought to serve as a safety buffer, maintaining cellular Ca^2+^ homeostasis in the event of a temporary intracellular Ca^2+^ overload, and promoting mitochondrial oxidative phosphorylation and ATP synthesis (Vasington and Murphy, 1962; Rizzuto et al., 1992). However, viral infection can lead to continuous Ca^2+^ accumulation and induce mitochondrial Ca^2+^ overload. Most of the mitochondrial Ca^2+^ overload comes from release from the ER, which is mainly because viral infection induces membrane contact between the ER and mitochondria (Vance, 2014). Mitochondria tightly associated with elements of ER can be isolated, and these membranes structures are frequently called mitochondria-associated membranes (MAMs) (Figure 1). IP_3_Rs are highly concentrated Ca^2+^ channels in the MAMs and play a central role that turn on/off Ca^2+^ release from ER stores (Mikoshiba and Hattori, 2000). Ca^2+^ released by ER is transported to the mitochondrial intermembrane space through the Ca^2+^ channel VDACs on the outer mitochondrial membrane (OMM) (Gincel et al., 2001), and the complicated MCU complex involved in Ca^2+^ transport through the inner mitochondrial membrane (IMM), and collaborative molecules such as cytosolic chaperone Grp75 are involved in regulation (Baughman et al., 2011; Drago et al., 2011; Poston et al., 2013). The molecular components of the uniporter comprise the pore-forming subunits MCU and dominant-negative regulator MCUb, together with calcium sensors mitochondrial calcium uptake (MICU)1, MICU2, and attachment essential MCU regulator (EMRE) (Raffaello et al., 2013; Sancak et al., 2013; Kamer and Mootha, 2015; Kamer, 2018). Apoptosis is closely related to the Ca^2+^ status of the mitochondria, these spatial contacts between the ER and mitochondria, are often used to function as viral targets, which triggers apoptosis by regulating the movement of Ca^2+^ between the ER and mitochondria (Csordás et al., 2006; Raffaello et al., 2016; Panda et al., 2021). For example, HCV, Enterovirus C (PV) and Enterovirus B trigger apoptosis through enhancing IP_3_R activity and promoting mitochondrial Ca^2+^ uptake by MCUs and VDACs (Gong et al., 2001; Brisac et al., 2010; Peischard et al., 2019). Mitochondrial Ca^2+^ efflux channels also play an important role in maintaining mitochondrial Ca^2+^ balance. Ca^2+^ efflux occurs through NCX and Ca^2+^/H^+^ antiporter (Khananshvili, 2013; Belosludtsev et al., 2019) (Figure 1).

Like IP_3_Rs, RyRs are responsible for regulating release of ER Ca^2+^ (Figure 1). Both IP_3_Rs and RyRs have similar tetramer structures and activation mechanisms, and are both activated by low Ca^2+^ levels and inhibited by high Ca^2+^ levels, so the low amount of Ca^2+^ released by ER further stimulates IP_3_Rs and RyRs activity (Fan et al., 2015). For example, HSV, HBV, HTLV-1 and IAV infections all lead to increased intracellular Ca^2+^, mainly due to abnormal release of ER Ca^2+^ caused by activation of IP3Rs (Hartshorn et al., 1988; Ding et al., 2002; Cheshenko et al., 2003; Xia et al., 2006). When ER Ca^2+^ storage release is increased, the ER calcium ATPase (SERCA pump) is activated and allows rapid reuptake of cytosolic Ca^2+^ by the ER (Courjaret and Machaca, 2014) (Figure 1). HCV core protein overexpressed in Huh7 cells induces ER Ca^2+^ depletion by impairing SERCA pump function (Benali-Furet et al., 2005). Depletion of intracellular Ca^2+^ storage also stimulates interaction between ORAI1 on the plasma membrane and STIM1 on the ER to mediate extracellular Ca^2+^ entry (Figure 1). In addition, some viroporins form channels on the ER that directly or indirectly increase ER permeability, leading to uncontrolled outflow of Ca^2+^ into the cytoplasm (Feng et al., 2002; Griffin et al., 2003; Peischard et al., 2019; Strtak et al., 2019).

In general, viral infection induces the formation of contact sites between MAMs and mitochondria, and the abnormal release of ER Ca^2+^ leads to mitochondrial Ca^2+^ overload and initiation of apoptosis.

### Mechanisms of Mitochondrial Membrane Permeabilization

The effect phase of apoptosis involves decreased mitochondrial membrane potential, respiratory chain uncoupling, mitochondrial permeability enhancement, and mitochondrial swelling and rupture (Hunter and Haworth, 1979). MPTP is the release channel of apoptotic molecules (such as Cyt C and AIF) and the transport channel of mitochondrial Ca^2+^, and its increased permeability is the decisive change in the early stage of apoptosis (Szalai et al., 1999). When viruses induce mitochondrial Ca^2+^ overload, accumulation of ROS and mitochondrial membrane potential dissipation, the opening of MPTP also releases Ca^2+^ from the mitochondria. For example, Murid betaherpesvirus 1 (Panel et al., 2019), JEV (Huang et al., 2016), IAV protein PB1-F2 (Chanturiya et al., 2004) and KHSV protein K7 (Feng et al., 2002) disrupt cytoplasmic Ca^2+^ levels and destroy mitochondria.

MPTP is a protein complex that connects the cytoplasm, OMM and IMM, intermembrane space of mitochondria and mitochondrial matrix, and is composed of component and regulatory molecules. The components include VDAC, adenine nucleotide translocase (ANT), and mitochondrial matrix protein cyclophilin D (Cyp D). Cyp D is also considered to be the key to MPTP opening, and is blocked by cyclosporine A (Kokoszka et al., 2004; Li et al., 2004; Baines et al., 2007) (Figure 4A). In recent years, some new molecules that may be involved in the formation of MPTP have been discovered, such as phosphate carrier, aspartate–glutamate carrier, ornithine–citrulline carrier and mitochondrial complex I on the electron transport chain (Fontaine et al., 1998; Chauvin et al., 2001). Some viral proteins locate in the mitochondrial membrane and interact directly with the MPTP to induce its opening (Jacotot et al., 2000; Chami et al., 2003).

ROS are effective activators of MPTP opening, which is induced by increased oxidative stress, and regulate Ca^2+^ channels in the ER and mitochondria. At the initiation stage of oxidative stress apoptosis, the transient reversible opening of MPTP increases and ROS accumulation causes damage to cells (Ma et al., 2011). MPTP continues to open irreversibly, causing mitochondrial swelling and OMM rupture, and proapoptotic proteins in the mitochondrial intermembrane are released into the cytoplasm and activate apoptotic responses. At the late stage of apoptosis, ROS are not cleared due to disorder of the antioxidant system in the mitochondria and cytoplasm. High concentrations of ROS trigger oxidative stress, resulting in mitochondrial membrane potential dissipation, mitochondrial oxidative damage, and finally induction of apoptosis (Chen et al., 2003; Lin and Beal, 2006; Stowe and Camara, 2009). In conclusion, viral infection causes abnormal release of ER Ca^2+^, accumulation of ROS, and excess opening of MPTP, which play a synergistic role in apoptosis.

## Ca^2+^ Signaling in Innate Immune and Antiviral Responses

Innate immunity is the first barrier of host defense against pathogenic microorganisms and endogenous stress responses, and it acts *via* different pattern recognition receptors (PRRs), including Toll-like receptors (TLRs), retinoic-acid-inducible gene I (RIG-I)-like receptors (RLRs), cytosolic DNA sensors, and nucleotide-binding oligomerization domain (NOD)-like receptors (NLRs), which cause the production of interferons (IFNs) and stimulate inflammatory cytokines after activation stimulated by microbial or cellular damage (Bowie and Unterholzner, 2008; Takeuchi and Akira, 2010; Liu et al., 2016). In addition, because Ca^2+^ signaling is involves in a variety of diseases, including viral infections, autoimmune diseases, and cancer, it represents an ideal target for PRRs. Conversely, intracellular Ca^2+^ signaling also modulates the activation of the PRR subfamily, facilitating activation of IFN regulatory factor (IRF) 3/7, initiating IFN-associated innate immune responses, and enhancing NF-κB-related inflammatory responses (Kong et al., 2021). In this section, we discuss the role of Ca^2+^ signaling in innate immune responses.

### Role of Ca^2+^ in TLR Signaling

Among the recognized PRRs, TLRs are the most extensive and oldest form of pathogen recognition, and can recruit multiple adaptor proteins to activate transcription factors, including NF-κB, activating protein (AP)-1, and interferon (IFN) regulatory factor (IRF) family members, to cause a further inflammatory reaction and IFN-dependent antiviral immune response (Nie et al., 2018). TLRs and Ca^2+^ signaling interact with and regulate each other. TLRs (including TLR2, TLR3, TLR4, TLR7, TLR8 and TLR9) have been demonstrated to participate in cytokine production, activation of immunocytes, inflammation, and antiviral innate responses by regulating Ca^2+^ signaling (Tauseef et al., 2012; De Dios et al., 2020; Zhao et al., 2020). In particular, TLR4, which recognizes bacterial lipopolysaccharides (LPSs), is widely reported to alter cytoplasmic Ca^2+^ levels and activates Ca^2+^ signaling by regulating various Ca^2+^ channels. For example, endotoxin activates transient receptor potential canonical (TRPC) channels to induce Ca^2+^ entry in endothelial cells, secondary to TLR4-induced diacylglycerol generation (Feske et al., 2012). TLR3, TLR7, and TLR8 are RNA sensors that recognize immune-stimulated RNA and initiate downstream signals that increase the production of inflammatory cytokines and type I IFN (IFN-I). These processes are still accompanied by extracellular Ca^2+^ influx or release of Ca^2+^ from the ER and activation of associated Ca^2+^ channels. Especially when HIV infects CD4^+^ T cells, TLR7 induces increased cytoplasmic Ca^2+^, sensitizing activated T cell 2 nuclear factor (NFATc2) through calcination, thereby promoting HIV replication (Dominguez-Villar et al., 2015). By contrast, Ca^2+^ signaling also modulates TLR signaling by affecting expression of different TLR molecules or controlling TLR activation in different ways. High Ca^2+^ upregulates mRNA expression of TLR3 and other dsRNA sensors to augment antiviral activity in epidermal keratinocytes (Yamamura et al., 2018). Extracellular Ca^2+^ influx by STIM1-operated Ca^2+^ channels transmit the information of TLR stimuli to initiate innate immune responses (Tang et al., 2017). In general, TLRs interact with intracellular Ca^2+^ signals through different molecular mechanisms. It is important to note that our understanding of TLR-mediated Ca^2+^-signal-dependent cellular functions in different pathological processes is still insufficient. In particular, the effect of TLRs on Ca^2+^ signal regulation in viral infection needs more investigation.

### Role of Ca^2+^ in RLR Signaling

RLRs are RNA sensor molecules that recognize the double-stranded RNAs (dsRNA), three of which are encoded in the human genome [RIG-I, melanoma differentiation-associated gene 5 (MDA5), and laboratory of genetics and physiology 2 (LGP2)], and enhance IFN production and play an important role in RNA virus infections (Yoneyama et al., 2005; Onomoto et al., 2021). RIG-I and MDA5 contain a tandem caspase recruitment domain, and interact with the mitochondrial antiviral signaling protein (MAVS) to activate IRF-3/7 and NF-κB, enhance IFN production, and promote transcriptional activation of proinflammatory cytokines (Yoneyama et al., 2015; Rehwinkel and Gack, 2020). Similarly, some data suggest that Ca^2+^ is a key regulator of the RLR pathway by regulating expression of many molecules involved in RLR signaling, or *via* regulation of Ca^2+^ channels. Studies have found that high levels of Ca^2+^ induce dsRNA sensors like MDA5 and RIG-I (Yamamura et al., 2018). In addition, the effects of some Ca^2+^ channels on RIG-I and MDA5 expression are related to Ca^2+^ signaling; for example, the Cav1.2 channel (a type of L-type Ca^2+^ channel) increases expression of RIG-I and MDA5 (Tammineni et al., 2018). Many reports have suggested that Ca^2+^ signaling plays an important role in the activation of RLR pathways during RNA virus infections. For example, IAV induces production of ROS to facilitate interaction of viral M2 protein with MAVS by increasing Ca^2+^ levels (Wang R. et al., 2019). Murine respirovirus can activate IRF3/7 by stimulating the ER to release Ca^2+^ through the RyR channel (Sermersheim et al., 2020). However, some studies have found that viruses recruited calcineurin, which inhibited TANK-binding kinase 1 (TBK1) phosphorylation and leading to reduced IFN-I production. Vesicular Stomatitis Virus (VSV) and HSV can restrict RLR-pathway-related antiviral innate immune response by targeting this mechanism (Huang et al., 2018). Pestivirus C infection can increase intracellular-Ca^2+^-level-induced autophagy through calcium/calmodulin dependent protein kinase 2 to suppress MAVS and decrease IFN-I production (Xie et al., 2021). Notably, the Ca^2+^ signaling pathway contributes to the regulation of RLR-mediated innate immunity, while there is no clear evidence that RLRs can control Ca^2+^ signaling. In addition, Ca^2+^ signaling can promote RLR-mediated innate immune response and reverse it in different viral infections. Therefore, it is important to illustrate the exact role of Ca^2+^ signaling in RNA virus regulation of RLRs in the future.

### Role of Ca^2+^ Signaling in the cGAS-STING Axis

Mammalian cells recognize double-stranded DNA (dsDNA) to produce type I interferons (IFNs), suggesting that cytosolic DNA sensing is the important mechanism by which the innate immune system detects pathogens (Stetson and Medzhitov, 2006; West et al., 2015; Erdal et al., 2017). Several cytosolic DNA sensors, including cyclic GMP–AMP synthase (cGAS), melanoma 2, and DNA-dependent IFN regulatory activator, have been identified as involved in immune responses. An increasing number of studies on the activation of type I IFNs response by viral infection have focused on the cGAS–STING axis, and recent studies have shown that Ca^2+^ and related signaling proteins regulate cGAS–STING (Mathavarajah et al., 2019). cGAS detects reverse transcription of retroviral RNA, aberrant release of viral DNA during infection, and damage to host genomic DNA or mitochondrial DNA (Gao et al., 2013; Sun et al., 2013; Kanneganti et al., 2015). Recognition of cytosolic DNA by cGAS results in the production of second messenger cGAMP that binds to stimulator of interferon genes (STING) (Ishikawa and Barber, 2008). Activation of STING interacts with TBK1, and then STING functions as a scaffold protein for TBK1 and IRF3 assembly to stimulate phosphorylation of IRF3 and nuclear translocation and induces the transcription of IFN genes (Fitzgerald et al., 2003; Tanaka and Chen, 2012).

The current study has demonstrated that STING has a Ca^2+^-binding site; when STING forms a homodimer, two ions are shared between the two monomers of the protein, so Ca^2+^ directly participates in the regulation of STING activation (Shu et al., 2012). STING interacts with some Ca^2+^ channels to regulate Ca^2+^ flux during its activation; for example, STING interacts with Ca^2+^ transporters VDAC to facilitate Ca^2+^ uptake by mitochondria. In the resting state, STING is located in the ER and binds Ca^2+^-sensing transmembrane protein STIM1 and interacts with SERCA (Lee et al., 2013; Srikanth et al., 2019). In addition, changes in cytosolic Ca^2+^ caused by viral infection facilitate STING activation. It has been found that BAPTA-AM (an extracellular Ca2+ chelator)-mediated Ca^2+^ depletion and ionomycin-mediated Ca^2+^ elevation suppress STING-mediated IFN-β production. The function of STING is also restricted when virus-infected cells are treated with the IP_3_R inhibitor 2-APB or SERCA inhibitors (Hare et al., 2015; Kwon et al., 2018; Mathavarajah et al., 2019). There is growing evidence that the cGAS–STING axis is one of the cellular pathways controlled by Ca^2+^ signaling, and the mechanism of this pathway regulating IFN-I response in microbial infection and viral diseases will be carefully studied in the future.

### Role of Ca^2+^ in Inflammatory Responses

The NLR family includes a variety of specific cytoplasmic sensors that detect invasive pathogens and endogenous danger signals. NOD-like receptor protein (NLRP)3 is one of the best-identified DNA sensors associated with inflammasomes in the NLR family, and is activated by invading pathogens or endogenous danger signals, leading to the formation of NLRP3 inflammasomes. The NLRP3 inflammasome is a multiprotein platform that includes NLRP3, apoptosis-associated speck-like protein (ASC) and pro-caspase-1, which leads to caspase-1-dependent secretion of proinflammatory cytokines IL-1β and IL-18 (Wen et al., 2013; Liu et al., 2018). The role of Ca^2+^ signaling in NLRP3 inflammasome activation has been widely reported. Lots of evidence show that Ca^2+^ from the extracellular environment, ER, and lysosome promote the formation and activation of NLRP3 inflammasome (Swanson et al., 2019; Li et al., 2021). For example, the cytosolic Ca^2+^ mediated by ion channels TRPA1 and TRPV1 facilitate the activation of the NLRP3 inflammasome (Wang M. et al., 2019); The release of the Ca^2+^ from ER through RyRs and IP_3_Rs are also observed to sensitize the NLRP3 inflammasome (Triantafilou et al., 2013a); Emanate from lysosomal Ca^2+^ stores regulate the production of pro-IL-1β by calcineurin, contributes to the activation of the NLRP3 inflammasome (Weber and Schilling, 2014). Until now, some data prove that viroporins enhance cytosolic Ca^2+^ by altering organelle membrane permeability, which promotes activation of NLRP3 inflammasomes. For example, Human rhinovirus B protein 2B targets ER and Golgi complex trigger Ca^2+^ release to stimulate NLRP3 inflammasome activation (Triantafilou et al., 2013b). Similarly, foot-and-mouth disease virus (FMDV) 2B protein as viroporins also promotes the flux of Ca^2+^, thereby stimulating NLRP3 inflammasome activation (Zhi et al., 2020). What’s more, severe acute respiratory syndrome coronavirus (SARS-CoV) envelope protein also activates the NLRP3 inflammasome by forming protein-lipid channels in ER/Golgi membranes to osmose Ca^2+^ (Nieto-Torres et al., 2015). So far, how intracellular Ca^2+^ stimulates NLRP3 activation is not fully understood. In the future, more investigations are needed to uncover the mechanisms by which Ca^2+^ mobilization induces NLRP3 inflammasome activation.

## Conclusion

Viruses make full use of Ca^2+^ signaling networks to facilitate entry, replication, assembly and export to establish persistent infection by altering Ca^2+^ homeostasis. Accumulation of pathological Ca^2+^ signals caused by viral infection can trigger cellular antiviral response and cell death. Therefore, Ca^2+^-signaling networks have become an important and effective target for the treatment of viral infections. Inhibitors or activators that act on different Ca^2+^ channels have potential as antiviral agents, which may help in the development of antiviral drugs. Ca^2+^, as a signaling messenger, is transferred between organelles and participates in the regulation of various cellular activities. Mitochondria are involved in many life activities including energy synthesis, cell death and immune response, understanding that Ca^2+^ regulates mitochondrial involvement in the immune response and cell death during viral infection is crucial. However, there are limited research data available on the molecular mechanisms of Ca^2+^ interaction with the Golgi complex, peroxisomes and lysosomes, these organelles can also be targeted and hijacked by viruses. Therefore, more work is warranted to illustrate the exact effect of Ca^2+^ on organelle signaling during viral infection. With improving understanding of Ca^2+^ signaling pathways and the development of Ca^2+^ signal monitoring technology, the effect of Ca^2+^ signal changes on cell fate in the process of viral infection will become an important topic.

## Author Contributions

CD and ZY conceived the review concept and drafted the article. YQ wrote the original draft and prepared figures. YS edited and reviewed the manuscript. All authors contributed to the article and approved the submitted version.

## Funding

This work was supported by the Shanghai Agriculture Applied Technology Development Program, China (G20180207), the National Natural Science Foundation of China (grant no. 32030108), and the Foundation of Key Laboratory of Veterinary Biotechnology (no. shklab202001).

## Conflict of Interest

The authors declare this research was conducted in the absence of any commercial or financial relationships that could be construed as a potential conflict of interest.

## Publisher’s Note

All claims expressed in this article are solely those of the authors and do not necessarily represent those of their affiliated organizations, or those of the publisher, the editors and the reviewers. Any product that may be evaluated in this article, or claim that may be made by its manufacturer, is not guaranteed or endorsed by the publisher.
